# Effect of Ultrasound on Dissolution of Polymeric Blends and Phase Inversion in Flat Sheet and Hollow Fiber Membranes for Ultrafiltration Applications

**DOI:** 10.3390/membranes15040120

**Published:** 2025-04-10

**Authors:** Gilberto Katmandú Méndez-Valdivia, María De Lourdes Ballinas-Casarrubias, Guillermo González-Sánchez, Hugo Valdés, Efigenia Montalvo-González, Martina Alejandra Chacón-López, Emmanuel Martínez-Montaño, Beatriz Torrestiana-Sánchez, Herenia Adilene Miramontes-Escobar, Rosa Isela Ortiz-Basurto

**Affiliations:** 1 Laboratorio Integral de Investigación en Alimentos, Tecnológico Nacional de México-Instituto Tecnológico de Tepic, Tepic 63175, Mexico; gikamendezva@ittepic.edu.mx (G.K.M.-V.); emontalvo@tepic.tecnm.mx (E.M.-G.); mchacon@tepic.tecnm.mx (M.A.C.-L.); headmiramonteses@ittepic.edu.mx (H.A.M.-E.); 2 Facultad de Ciencias Químicas, Universidad Autónoma de Chihuahua, Chihuahua 31125, Mexico; 3 Departamento de Medio Ambiente y Energía, Centro de Investigación en Materiales Avanzados, Chihuahua 31136, Mexico; guillermo.gonzalez@cimav.edu.mx; 4 Centro de Innovación en Ingeniería Aplicada, Departamento de Computación e Industrias, Universidad Católica de Maule, Talca 3460000, Chile; hvaldes@ucm.cl; 5 Maestría en Ciencias Aplicadas, Unidad Académica de Ingeniería en Biotecnología, Universidad Politécnica de Sinaloa, Mazatlan 82199, Mexico; emartinez@upsin.edu.mx; 6 Unidad de Investigación y Desarrollo de Alimentos, Tecnológico Nacional de México-Instituto Tecnológico de Veracruz, Veracruz 91897, Mexico; beatriz.ts@veracruz.tecnm.mx

**Keywords:** green solvent, cellulose acetate, ultrasound-assisted phase inversion, sono-extruder

## Abstract

In seeking alternatives for reducing environmental damage, fabricating filtration membranes using biopolymers derived from agro-industrial residues, such as cellulose acetate (CA), partially dissolved with green solvents, represents an economical and sustainable option. However, dissolving CA in green solvents through mechanical agitation can take up to 48 h. An ultrasonic probe was proposed to accelerate mass transfer and polymer dissolution via pulsed interval cavitation. Additionally, ultrasound-assisted phase inversion (UAPI) on the external coagulation bath was assessed to determine its influence on the properties of flat sheet and hollow fiber membranes during phase inversion. Results indicated that the ultrasonic pulses reduced dissolution time by up to 98% without affecting viscosity (3.24 ± 0.06 Pa·s), thermal stability, or the rheological behavior of the polymeric blend. UAPI increased water permeability in flat sheet membranes by 26% while maintaining whey protein rejection above 90%. For hollow fiber membranes, UAPI (wavelength amplitude of 0 to 20%) improved permeability by 15.7% and reduced protein retention from 90% to 70%, with MWCO between 68 and 240 kDa. This report demonstrates the effectiveness of ultrasonic probes for decreasing the dissolution time of dope solution with green cosolvents and its potential to change the structure of polymeric membranes by ultrasound-assisted phase inversion.

## 1. Introduction

Filtration using polymeric membranes has a broad range of applications in science and industry due to its low energy consumption, continuous separation capability, and ease of scalability, among other advantages [1,2]. The base polymer of membranes defines key properties, such as hydrophobicity, surface charge, and selectivity [3]. Most polymeric membranes are made from polymers like polyethersulfone (PES) [4], polysulfone (PSF) [5], polyvinylidene fluoride (PVDF) [6], or polypropylene (PP) [7], as well as cellulose [8] and its derivatives, such as cellulose acetate (CA) [9]. CA membranes offer distinct advantages, including high hydrophilicity, which facilitates the separation of water-soluble compounds, maintains significant flux, and reduces fouling propensity. These benefits are complemented by good mechanical strength and thermal stability [10,11,12]. Our focus is on producing membranes made from cellulose acetate derived from natural sources. Several studies have investigated the synthesis of cellulose triacetate from various natural biomass sources, highlighting its potential as a valuable material. Meireles et al. [13] successfully produced cellulose acetate from mango seeds, achieving a high degree of substitution (DS) of 2.65. Similarly, Das et al. [14] synthesized cellulose acetate from rice husks using various methods, obtaining a notable DS. Another study demonstrated that solid waste from the olive industry could serve as an important source of cellulose powder and its derivatives [15]. Soto-Salcido et al. [16] focused on extracting cellulose acetate from agave bagasse for film production, showcasing the versatility of this biomass source. Additionally, Villanueva-Solís et al. [17] worked with oak sawdust to obtain cellulose acetates, further broadening the range of potential feedstocks for cellulose derivative synthesis.

The primary method for the elaboration of ultrafiltration polymeric membranes is non-solvent-induced phase separation (NIPS) [18], using a homogeneous solution (dope) composed of the base polymer and polymeric additives, such as methylcellulose, polyvinyl pyrrolidone (PVP), and polyethylene glycol (PEG); this could dissolve in water during the NIPS process (a pore-forming agent) or remain in the membrane matrix (hydrophilic and antifouling agent) or could have both roles [19]. The most commonly used solvents for preparing the dope solution with cellulose acetate by NIPS include N-methyl-2-pyrrolidone (NMP), dimethylacetamide (DMAc), dimethyl sulfoxide (DMSO), and N,N-dimethylformamide (DMF) [20]. However, these solvents pose significant health and environmental risks. Consequently, replacing them with non-toxic, biodegradable green solvents is essential [21]. Candidates for green solvents include γ-butyrolactone, γ-valerolactone, methyl acetate, triethyl phosphate, and glycerol derivatives like glycerol diacetate (diacetin) and glycerol triacetate (triacetin) have been suggested [22,23,24,25]. Unfortunately, green solvents often dissolve polymer blends and “dope” solutions less efficiently, as their Hildebrand and Hansen solubility parameters are significantly lower than those of conventional solvents. This means that the solubility parameters of green solvents and polymers are more different, resulting in lower cohesion energy (thermodynamic affinity) [24,25,26].

Various methods, including ultrasound, can increase the polymer’s dissolution rate in the solvent under standard temperature and pressure conditions. High-power ultrasound produces mechanical, acoustic, thermal, and chemical effects through cavitation, enhancing polymer-solvent contact and accelerating the dissolution rate [27,28]. The mechanical effects of cavitation generate high temperatures (up to 1000 °K), localized shear forces, microjets (up to 100 m/s), and shockwaves (up to 30 MPa) [29]. Cavitation involves the violent collapse of bubbles, resulting in molecular fragmentation, ultrasonic diffusion, and enhanced mass transfer [30], which accelerates the dissolution of homogeneous polymer blends for membrane production [31]. This is particularly important given cavitation’s reported effects on viscosity, polymerization, and potentially irreversible polymer chain degradation [32,33,34]. The ability of ultrasonic baths to dissolve polymers, disperse additives in dope solutions, and degas them to avoid imperfections during membrane manufacturing has been reported. Table 1 presents the effects of the US on the dissolution of polymeric mixtures and the production of flat membranes by US-assisted phase inversion (UAPI) in an ultrasonic bath. However, it has also been reported that ultrasonic probes can depolymerize cellulose, causing a reduction in viscosity and alteration of solution properties [35]. It is, therefore, of interest to verify that applying a high-power ultrasonic probe to reduce the dissolution time of polymeric mixtures does not affect their properties that could change the properties of the membrane.

The dope solution is spread or extruded by NIPS, inducing phase inversion and membrane formation into a non-solvent coagulation bath, typically water [42]. The active layer structure depends on system thermodynamics [43]. Studies using ultrasound in the dope solution and membrane phase inversion with other materials are limited and presented in Table 1. Therefore, studying the impact of ultrasound on a polymer blend based on cellulose acetate, where DMF is partially replaced by diacetin, is essential for evaluating rheological and thermogravimetric properties. This is particularly important given cavitation’s ultrasound probe-reported effects on viscosity, polymerization, and potentially irreversible polymer chain degradation [32,34].

This study aims to (1) evaluate the use of a high-power ultrasonic probe to dissolve a polymer blend containing CA, PEG, DMF, and diacetin to reduce dissolution time and verify the stability of the properties of the polymer mixture. And (2) assess the ultrasound-assisted phase inversion on the membrane structure, permeability, and changes in static (structure, MWCO) or dynamic (permeability and selectivity) properties of flat sheet and hollow fiber membranes for ultrafiltration applications.

## 2. Materials and Methods

### 2.1. Materials

The polymeric mixture was prepared using commercially available CA (acetyl content 39.8 wt%, Mw = 30,000 g/mol, Sigma Aldrich, St. Louis, MO, USA) and PEG (PEG 6000, Mw = 6000 g/mol, Sigma Aldrich, St. Louis, MO, USA) as a polymeric additive for membrane pore formation. Two solvents were used for polymer dissolution: diacetin (CH_3_COOCH_2_)_2_CHOH (50% technical-grade diacetin, Mw = 176.17 g/mol, Sigma Aldrich, St. Louis, MO, USA) as a green solvent and DMF (Mw = 73.09 g/mol, J.T. Baker, Phillipsburg, NJ, USA) as a cosolvent.

### 2.2. Dissolution of the Polymeric Mixture by Stirring and Ultrasound

**Figure A1:**
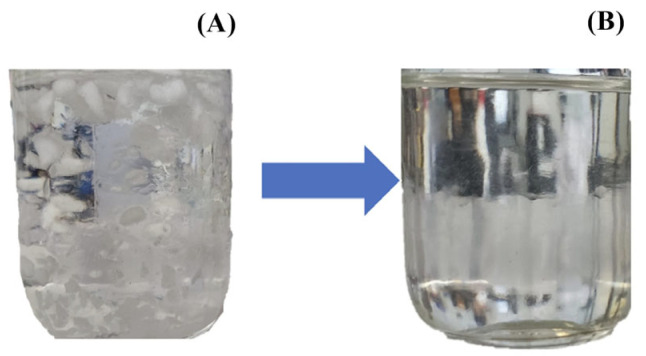
Dope solution: (**A**) with agglomerates; (**B**) total dissolution of polymers.

Five treatments were conducted to compare dissolution times. For stirring-based dissolution, a solution containing 12.5% CA (previously dried at 100 °C for 24 h), 42.25% DMF, 42.25% diacetin, and 5% PEG was heated to 55 °C until complete dissolution was observed. A factorial design was applied for ultrasound-assisted dissolutions, evaluating two methods using identical proportions and two ultrasonic amplitudes. The first method involved dissolving PEG with the solvents before adding CA, while the second dissolved all components simultaneously. A stainless-steel ultrasonic probe with a diameter of 0.5 and a length of 2.5 inches was used, connected to a high-power (700 W) ultrasonic generator QSonica-700 (Newtown, CT, USA) operated at 20 kHz for both methods, with amplitudes of 50% and 100%, applied in pulsed intervals of 30 s/min until total dissolution of polymer agglomerates was achieved (Figure A1).

### 2.3. Characterization of the Polymeric Mixture

#### 2.3.1. Viscosity and Rheological Behavior

As Miramontes-Escobar [1] reported, apparent viscosity was determined using a Discovery DHR 1 hybrid rheometer (TA Instruments, New Castle, DE, USA) with a 40 mm serrated cone-plate geometry at 25 °C. Dynamic rheological studies were performed on mixtures dissolved via ultrasound, involving a strain sweep from 1% to 100% deformation at a fixed frequency within the linear viscoelastic region. Angular frequency sweeps from 1 to 100 rad/s were conducted to evaluate viscoelastic behavior by monitoring the storage modulus (G′) and loss modulus (G″). Flow behavior was assessed by measuring viscosity as a function of shear rate from 1 to 100 s^−1^.

The flow behavior was analyzed using the Cross Equation (1) [44].

(1) $$\eta (\dot{\gamma \;})=\eta_{0}\frac{\eta_{\infty }-\eta_{0}}{1-{\lambda \dot{\gamma }}^{n}}$$

where $\eta (\dot{\gamma \;})$ represents apparent viscosity (Pa·s), $\eta_{\infty }$ is viscosity at infinite shear rate (Pa·s), $\eta_{0}$ is viscosity at zero shear rate (Pa·s), *λ* is the relaxation time (s), *n* is the flow index (dimensionless), and $\dot{\gamma \;}$ is the shear rate (s^−1^).

#### 2.3.2. Calorimetric Analysis of Polymeric Blends

Thermogravimetric analysis (TGA) was performed using a TGA-550 instrument (TA Instruments, New Castle, DE, USA) with sample weights ranging from 15 to 20 mg. Temperature was ramped from 25 °C to 800 °C at 10 °C/min under a nitrogen atmosphere [45].

Differential scanning calorimetry (DSC) was carried out using a DSC-250 instrument (TA Instruments, New Castle, DE, USA) with 10 mg samples. Temperature ranged from 25 °C to 400 °C at a heating rate of 10 °C/min under nitrogen.

### 2.4. Membrane Fabrication

#### 2.4.1. Ultrasound-Assisted Flat Sheet Membranes

**Figure 1:**
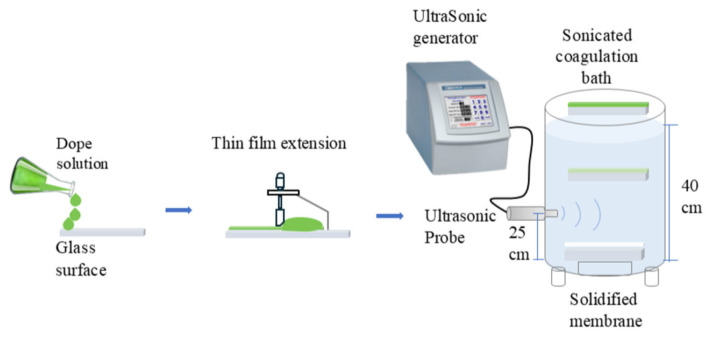
Diagram of flat membrane fabrication by ultrasound-assisted phase inversion.

**Figure A2:**
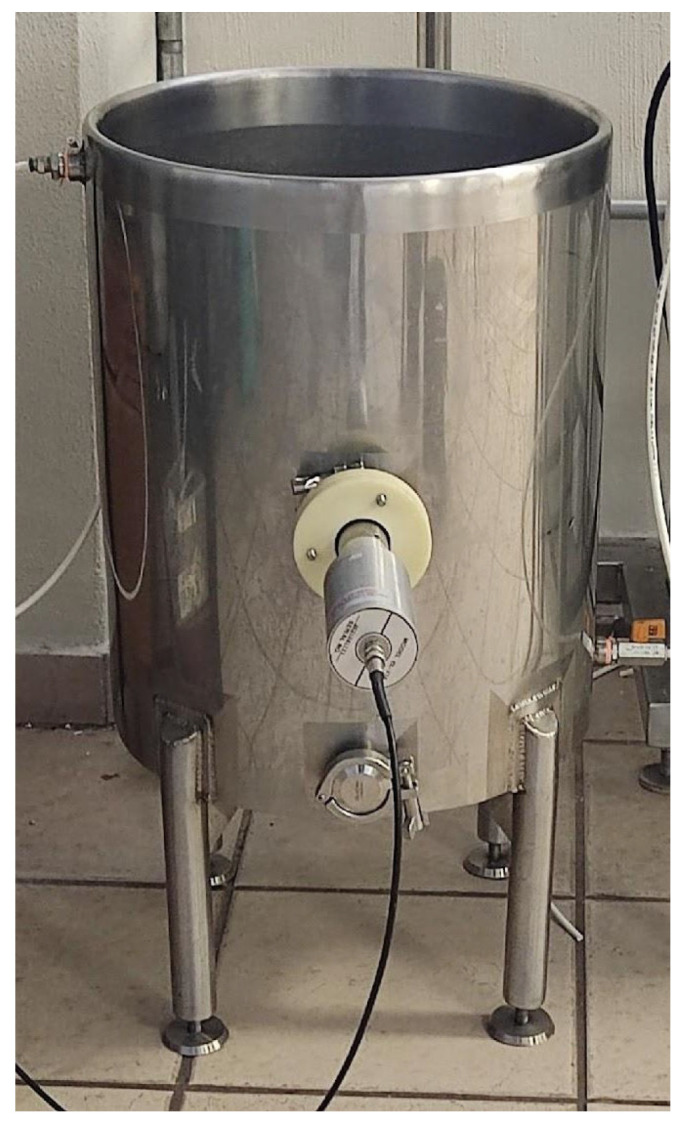
Phase inversion tank coupled with an ultrasonic probe.

Membranes were produced using the non-solvent-induced phase separation (NIPS) method (Figure 1), following the procedure by Ballinas-Casarrubias et al. [46], with modifications for ultrasound-assisted phase inversion. Approximately 20 mL of dope polymeric mixture solution was deposited on the upper end of a glass plate (14 × 19 cm); the solution was immediately spread using a film extensor model 3580 (Elcometer, Manchester, UK) calibrated to 150 µm. The glass plate with the extended film of 150 µm was quickly placed on the surface of the sonicated coagulant bath (with water as a nonsolvent) for induced phase separation and membrane structure formation. The glass plate was immersed by gravity (in 1–2 s) in a stainless steel tank (40 cm diameter, 55 cm height, with a double jacket for temperature control at 25 °C) filled with 40 L of filtered water (non-solvent). The coagulation tank (Figure A2) contained 25 cm from the bottom a stainless-steel ultrasonic probe with a diameter of 0.5 and a length of 2.5 inches, connected to a high power (700 W) ultrasonic generator QSonica-700 (Newtown, CT, USA). A factorial design was applied, evaluating three ultrasonic amplitudes (25, 50, and 75%) at different times (1, 3, and 5 min). In all cases, the ultrasound was turned on just before spreading the soap solution film on the glass; the time was counted from the moment the glass was deposited on the surface of the coagulant bath. The US probe allows the glass plate to reach the bottom of the external coagulant tank. The membranes were subjected to two washes (24 h each), the first with water and the second with 30% glycerol, to prevent structural collapse. They were dried at 40 °C before being stored in plastic bags until characterization.

#### 2.4.2. Ultrasound-Assisted Hollow Fiber Membranes

**Figure 2:**
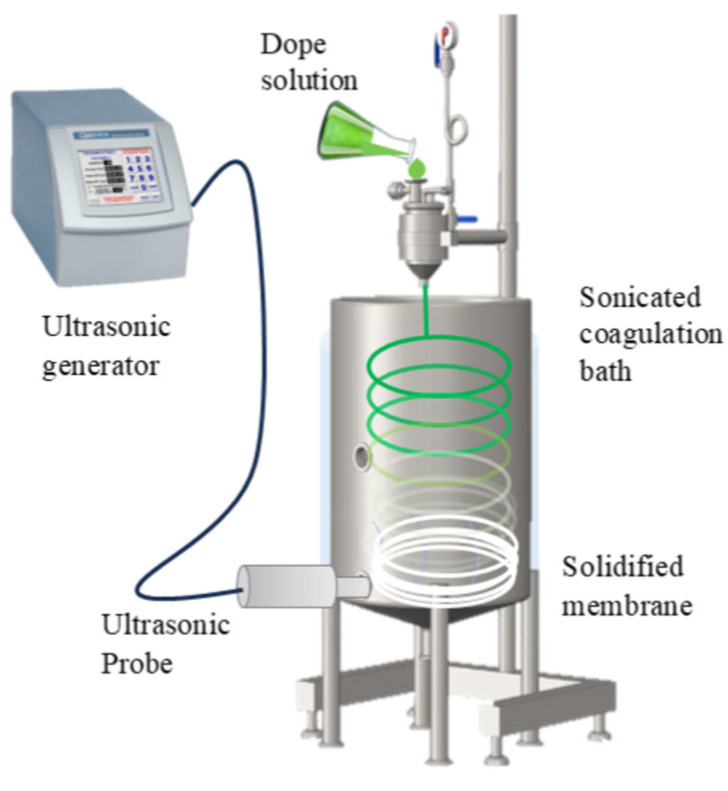
Graphic representation of ultrasound-assisted phase inversion in the elaboration of hollow fiber membranes.

**Figure A3:**
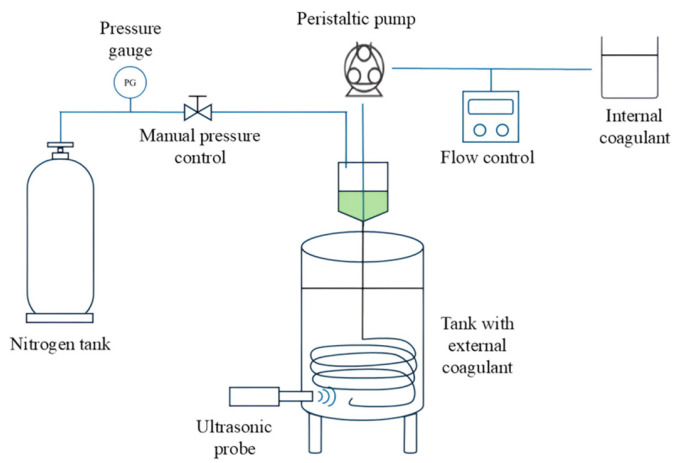
Graphic representation of the sono-extrusion pilot used for the elaboration of hollow fiber membranes.

Hollow fiber membranes were prepared following Torrestiana-Sánchez et al. [47], with modifications mainly in ultrasound-assisted phase inversion (Figure 2). Twenty-five milliliters of the dope polymeric mixture liquid were placed in a sono-extruder (designed in the laboratory, see Figure A3) with internal and external coagulation temperatures set to 25 °C. The air gap was fixed at 15 cm, internal coagulant flow at 15 mL/min, and extrusion pressure at 0.5 bar. A one-way statistical design was applied, evaluating three ultrasonic amplitudes (5, 10, and 20%) with the ultrasonic probe (on during all the extrusion) placed 50 cm away. After extrusion, fibers were submerged in water for 24 h, in glycerol for 24 h to prevent structural collapse, and dried for 24 h at 40 °C. Membranes were stored in bags for subsequent characterization.

### 2.5. Characterization of the Manufactured Filtration Membranes

#### 2.5.1. Internal Structure of the Membranes

The methodology described by Terrazas-Bandala et al. [48] was followed to determine the internal structure of the produced membranes. The membranes were exposed to liquid nitrogen, fractured, and coated with a thin gold layer using a Denton Desk-II Gatan (Moorestown, NJ, USA) coating system. Micrographs were captured at 10 kV using a scanning electron microscope (SEM SU3500, Hitachi High-Tech, Tokyo, Japan) in a cross-sectional view at the Advanced Materials Research Center.

#### 2.5.2. Filtration Performance (Flux and Retention)

The filtration performance analysis was conducted following the methodology of Rahimpour and Madaeni [49] with minor modifications. A 2% whey protein solution was filtered at room temperature for 60 min through all obtained membranes. Permeate samples were collected every 5 min, and turbidity was measured using a Turbidimeter HACH (Model 2100Qis, HACH COMPANY, Guiping Road, Shanghai, China).

The flux (*J*) was calculated using Equation (2).

(2) $$J=\frac{Q_{p}}{A\Delta P}$$

where $Q_{p}$ represents the permeate flow (L), *A* is the membrane surface area (m^2^), and *ΔP* denotes the transmembrane pressure (bar). The results were expressed in L·h^−1^·m^−2^.

Protein retention capacity (*R*, %) was determined using Equation (3).

(3) $$R=1-\frac{{NTU}_{p}}{{NTU}_{f}}\times 100$$

where *NTU_p_* is the particle count in the permeate, and *NTU_f_* is the particle count in the feed solution.

The Volume Reduction Factor (*VRF*) for different membranes was evaluated using Equation (4).

(4) $$VRF=\frac{V_{0}}{V_{f}-V_{0}}$$

where *V*_0_ is the initial sample volume (mL), and *V_f_* is the filtrate volume (mL) [50].

#### 2.5.3. Protein Retention by Electrophoretic Analysis

The electrophoretic analysis followed Laemmli’s method [51]. A stacking and separating gel (4% and 12% polyacrylamide, respectively) with sodium dodecyl sulfate (SDS) (Sigma Aldrich, St. Louis, MO, USA) was prepared. T0-A0 and T5-A75 permeate flat membrane samples were diluted (1:1) in the sample buffer and boiled for 3 min. Aliquots of 10–15 μL at a 2 mg/mL concentration were loaded onto the gel. Electrophoresis was performed using Tris-HCl buffer (Sigma Aldrich, St. Louis, MO, USA) (0.025 M, pH 8.8), glycine (Sigma Aldrich, St. Louis, MO, USA) (0.192 M), and SDS (0.1%) at 150 V for 30 min and 200 V for 50 min. The gel was stained with Coomassie Brilliant Blue R-250 (0.1% *w*/*v*) (Sigma Aldrich, St. Louis, MO, USA) in methanol-acetic acid (40% and 10% *v*/*v*) (Sigma Aldrich, St. Louis, MO, USA) for 24 h and destained in the same solution. Molecular weights were determined by applying five μL of a standard compared to a molecular weight marker (10–250 kDa) (SDS-PAGE Standards, Bio-Rad, Hercules, CA, USA).

#### 2.5.4. MWCO Determination by HPLC

The molecular weight cut-off (MWCO) in hollow fiber membranes was determined by HPLC. The proteins in defatted and lyophilized samples (So and permeated solutions) were determined following the methodology reported by González-Felix et al. [52] with some modifications. A Varian™ Pro Star (Varian Inc., Walnut Creek, CA, USA) high-pressure liquid chromatography equipped with a diode array detector (DAD) and Galaxy™ software version 1.9.302.952 was used for the analysis. Each sample was dissolved (20 mg/mL) in a mobile phase of 150 mM sodium phosphate buffer, pH 7, at 25 °C. A peptide (catalog 151-1901; BIORAD, Hercules, CA, USA) containing five known MW compounds (thyroglobulin, 670 kDa; gamma-globulin, 158 kDa; ovalbumin, 44 kDa; myoglobin, 17 kDa; and vitamin B12, 1.35 kDa) was used. A 20 µL aliquot was injected at an isocratic flow of 0.4 mL/min in a size exclusion column (Bio Sec-5^TM^, 4.6 x 300 mm; Agilent, Santa Clara, CA, USA), and the absorbance was monitored at a wavelength of 254 nm. The membrane’s molecular weight cut-off (MWCO) determination was performed by comparing the area under the curve determined in the chromatogram of the fed solution (So) and the 10% in the permeate obtained from each of the membranes.

### 2.6. Statistical Analysis

The results were analyzed using ANOVA with a significance value of *p* < 0.05 with Statistica v12 software. All analyses were performed in triplicate.

## 3. Results and Discussion

### 3.1. Dissolution of Polymer Mixture by Agitation and Ultrasound

#### 3.1.1. Dissolution Time and Viscosity of Polymer Blends

The dissolution time (min) and apparent viscosity (Pa·s) for different ultrasound intensities (W/cm^2^) are presented in Table 2. Ultrasound intensity, which measures energy applied per unit area, increases with amplitude, affecting dissolution efficiency. Various ultrasound intensities were used for comparative analysis.

The use of ultrasound reduced the effective dissolution time of the polymer blend from two days (48 h) under stirring to 35 min using ultrasonic pulses with an intensity between 55 and 60 W/cm^2^, regardless of the dissolution order, and to 15 min at an intensity between 118 and 123 W/cm^2^. This reduction is attributed to increased temperature and the effects of cavitation, such as acoustic streaming, microstreamers, microjets, and microstreaming, which enhance physical agitation and collisions within the container, generating micro vortices that promote improved mass transfer and diffusion. This process enables better solvent penetration into the polymers, significantly reducing dissolution time [53]. Other researchers reported similar findings. Lan et al. [31] used ultrasound at 20 to 75 W and 110 °C for 5 to 20 min to dissolve 2% cellulose in an ionic liquid (C4mim) Cl, reducing the dissolution time from 190 to 60 min. The improvement was attributed to ultrasound effects, such as cavitation and mechanical impact, which enhanced mass transfer between the ionic liquid and cellulose. Despite Ávila-Orta et al. [54] noting that increased ultrasonic intensity affects polymer viscosity, the apparent viscosity of the different samples in this study showed no significant differences (*p* < 0.05) compared to non-sonicated samples, indicating the polymer blend’s stability under applied ultrasonic intensities.

#### 3.1.2. Rheological Behavior of Polymer Blends

**Figure 3:**
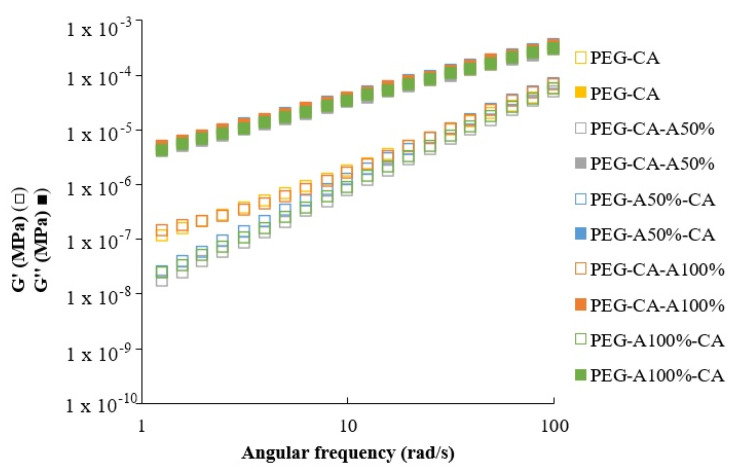
Effect of ultrasound on the polymer blend. Storage modulus G′ (□) and loss modulus G″ (■).

Frequent sweep tests with 25% deformation were conducted on all mixtures to confirm that ultrasound did not alter the polymer blend. Figure 3 shows storage modulus (G′) and loss modulus (G″) as functions of angular frequency.

The results showed that G″ predominated over G′, a characteristic of viscoelastic liquids [55]. G′ represented the energy stored within the blend during deformation, which increased with more intense interactions, while G″ represented dissipated energy as heat during deformation, allowing the most fluidity [56]. The predominance of G″ over G′ was primarily due to the compound’s functionality, where cellulose acetate’s -COCH_3_ groups prevent interaction with PEG’s OH-groups, reducing intermolecular interactions; consequently, the blend stored less energy, making G′ lower than G″ [57]. Additionally, G″ increased with rising angular frequency, reflecting increased friction between polymers, proportional to viscosity [58]. This behavior was consistent in all sonicated samples, confirming that ultrasound enhanced mass transfer and accelerated polymer dissolution without breaking functional groups or altering the blend’s behavior [28].

**Figure 4:**
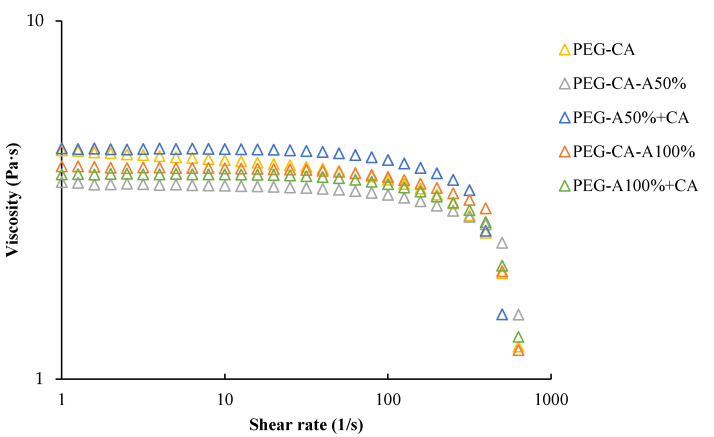
Dope solutions’ viscosity vs. shear rate curve for the treatments.

To determine the viscosity behavior under applied shear stress (Newtonian or Non-Newtonian), a flow curve (see Figure 4) was generated for the different treatments. Flow curves were used to examine the viscosity behavior under shear stress. Comparing sonicated and stirred blends revealed Newtonian behavior at shear rates below 100 s^−1^, while high rates showed a viscosity decrease, indicating shear-thinning behavior.

To confirm this without sample degradation, data were fitted to the Cross model, which accounted for Newtonian and shear-thinning behaviors. This polymer viscosity model allows the description of a Newtonian fluid, a shear-thinning fluid, or a shear-thickening fluid based on the parameter “n” [59].

Table 3 presents the fitted model constants using the TRIOS version 3 software (TA Instruments, New Castle, DE, USA). The viscosity trend $\eta_{0}\;$>${\;\eta }_{\infty }$ matched Figure 4, where initial viscosity values decreased with shear rate. Relaxation time (λ) values indicated low relaxation times for all blends, suggesting uniform recovery after stress removal [60]. All blends exhibited shear-thinning behavior (n < 1), confirming that viscosity decreased with increasing shear rate; this confirmed the observation in Figure 4, where an increase in shear rate tended to result in a decrease in apparent viscosity [61]. The Cross model confirmed shear-thinning behavior with low relaxation times, which is favorable for hollow fiber membrane extrusion processes due to enhanced spinning properties [62]. Overall, ultrasound-treated blends exhibited rheological behavior comparable to stirred blends, confirming no adverse effects on the polymer mixture.

#### 3.1.3. Calorimetric Analysis: TGA and DSC of Polymer Blends

**Figure 5:**
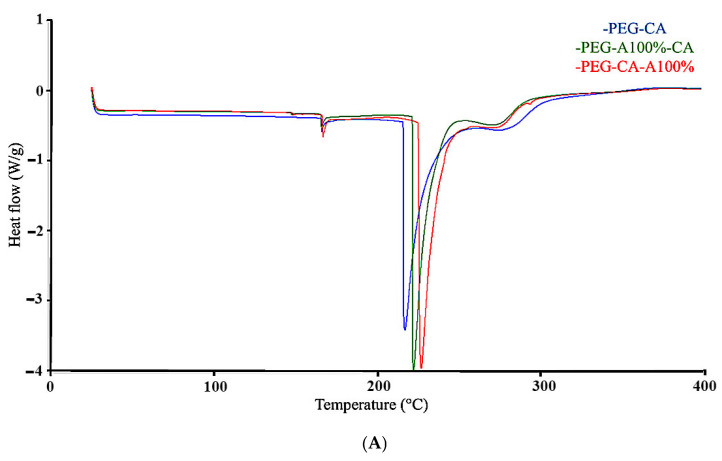

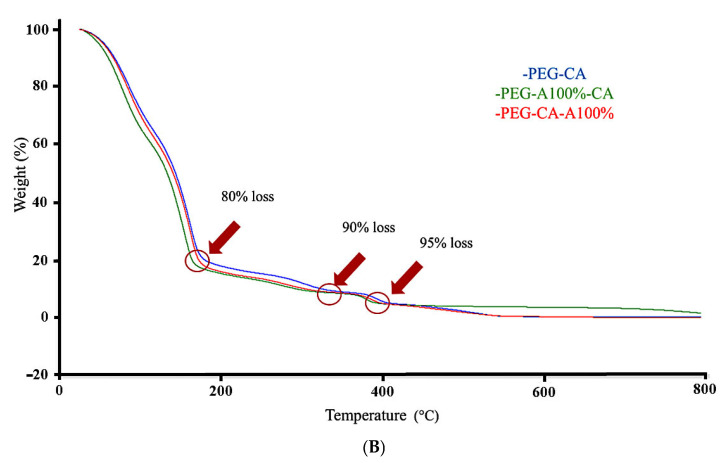
(**A**) Differential scanning calorimetry (DSC) curves and (**B**) thermogravimetric analysis (TGA) curves for stirred and sonicated polymer blends to be used for the membrane’s preparation.

Calorimetric analyses were performed on polymer blends treated with maximum ultrasonic intensity (120 W/cm^2^) to assess molecular stability (see Figure 5). DSC curves (Figure 5A) revealed two endothermic transitions for all samples (Table 4). The first transition corresponded to the glass transition temperature (T_g_), with similar values across blends: PEG-CA (165.94 °C), PEG-CA-100% (166.79 °C), and PEG-100%-CA (165.66 °C). Statistical analysis showed no significant effect (α < 0.05) on T_g_, confirming that ultrasound did not alter intermolecular interactions affecting T_g_. Vinodhini et al. [63] reported a T_g_ of 165 °C for chitosan-CA-PEG-DMF blends, consistent with this study’s findings, confirming minimal ultrasound impact on polymer chain arrangement. The second DSC transition (Figure 5A) represents the melting temperature (T_m_). T_m_ values varied slightly among blends: PEG-CA (216.61 °C), PEG-CA-100% (226.58 °C), and PEG-100%-CA (221.82 °C), suggesting ultrasound-induced polymer chain reorganization, increasing density and thermal stability [64]. Rodrigues et al. [65] reported a T_m_ close to 225 °C for commercial cellulose acetate, aligning with these results and indicating ultrasound promoted structural reorganization without degradation. On the other hand, TGA (Figure 5B) further evaluated polymer chain integrity and transition temperatures (Table 4), confirming ultrasound effects without compromising polymer stability. In addition to the DSC analysis, thermogravimetric analysis (TGA) was performed (Figure 5B) to study the polymeric chains and evaluate potential degradation at the transition temperature (Table 4), which may have been influenced by ultrasound. The first weight loss (80%) of the PEG-CA mixture occurred at 178 °C, whereas the mixtures subjected to ultrasound exhibited lower temperatures: 171 °C for the PEG-CA-100% sample and 163 °C for the PEG-100%-CA sample. This phenomenon is associated with the solvents, which have evaporation temperatures of 99 °C and 65 °C, respectively, values lower than the polymer degradation temperatures. The second weight loss (90%) was linked to the thermal resistance of PEG and CA, occurring at 320 °C for PEG-CA, 300 °C for PEG-CA-100%, and 290 °C for PEG-100%-CA. During the third loss (95%), carbonization was observed at 404, 396, and 395 °C, respectively. These differences, particularly in the second weight loss, suggested that ultrasound might have caused chain breakage, primarily due to homolytic scissions in polymeric chains and solvent oncolysis. These reactions promoted radical formation, which impacted the thermal stability of the polymeric mixture, causing the observed decrease [34]. However, previous studies [66,67] indicated that cellulose acetate remained stable between 260 °C and 450 °C, suggesting that ultrasound did not significantly affect the thermal stability of this polymer.

### 3.2. Effect of Ultrasound on the Elaboration of Flat Membranes

#### 3.2.1. Flat Membranes Internal Structure Micrographs

**Figure 6:**
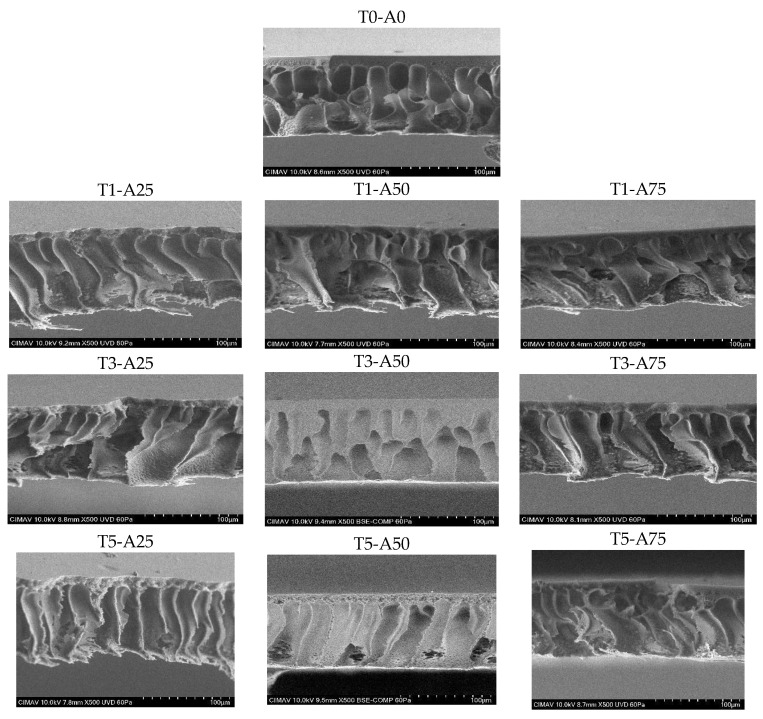
Micrographs evidencing the ultrasonic effect on the internal structure of flat membranes. T: time (ultrasound application in minutes); A: ultrasound amplitude (%).

Cross-sectional micrographs of flat membranes elaborated by ultrasound-assisted phase inversion under different US amplitudes (Figure 6) demonstrated that this method facilitated the formation of enlarged pores or anisotropic structures. This structure consisted of a dense layer providing membrane selectivity, followed by an extension of pores known as “finger-like” structures, which improve permeability. Membranes subjected to 25% and 50% US amplitudes for 5 min exhibited this structure. Huan et al. [38] showed that when flat PVDF membranes were produced using an ultrasonic bath with a power less than 60 W during phase inversion, macropore formation was reduced, and the tendency for finger-like structures with greater water flux, high porosity, and sizeable mean pore size of membranes was enhanced. This highlights that the applied power defines finger-like pore structures or teardrop-like voids.

Internal membrane structures exhibited more uniform cavity extensions at longer times and lower amplitudes, as seen in membrane T5-A25. However, increasing the amplitude caused more significant tortuosity in the support structure, observed in membranes treated at 75% amplitude. This effect was attributed to ultrasonic intensity generating higher vibrations and microjets that modified the structure, promoting irregular cavity formation. Furthermore, ultrasound enhanced mass transfer between solvents and water, accelerating phase inversion and solidification. This rapid solidification prevented pore-forming additives from creating finger-like structures in the support layer, altering the internal architecture [36].

#### 3.2.2. Whey Protein Filtration Capabilities of Flat Membranes

**Figure 7:**
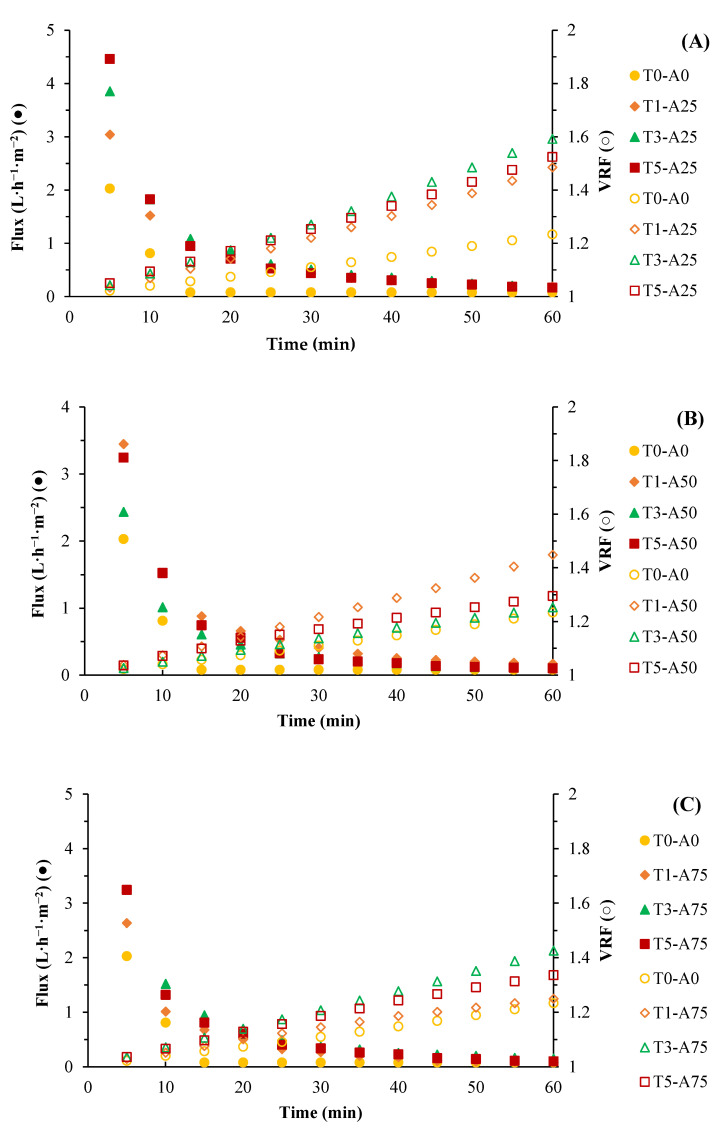
Performance and volumetric reduction factor (VRF) during whey protein filtration with flat membranes subjected to different ultrasonic amplitudes. T: time (ultrasound application in minutes), percentage of applied ultrasonic amplitude (**A**) 25 (**B**) 50 (**C**) 75 %. VRF (□). Flux (●).

The permeability behavior of membranes in a stirred tank with frontal filtration of a 2% whey protein solution at 1.5 bar pressure is illustrated in Figure 7. Initial permeability decline, characteristic of this technology, resulted from fouling layer formation on the membrane surface due to protein and ion (calcium, sodium, and zinc) adsorption. The flux reduction and consequently the permeability decrease correlated with an increasing volumetric reduction factor (VRF). Higher sodium concentrations increased protein size, exacerbating fouling [68]. However, membranes treated with ultrasound exhibited higher flux than untreated membranes (T0-A0).

Ultrasonically treated membranes exhibited permeability increases from 18.43% to 96.59%, while VRF increased from 4.46% to 15.14%, with significant differences (*p* < 0.05). This suggested that ultrasound-induced internal structural changes, such as increased extension pores and reduced macropores in the support layer. Increases in filtration performance in protein separation have been reported by reducing the tortuosity of the membrane due to the reduction in protein retention in the internal structure [69].

To assess whether increased permeability compromised molecular retention, turbidity reduction was measured using nephelometric turbidity units (NTU) (Table 5). The initial solution had a turbidity value of 903 NTU.

**Figure A4:**
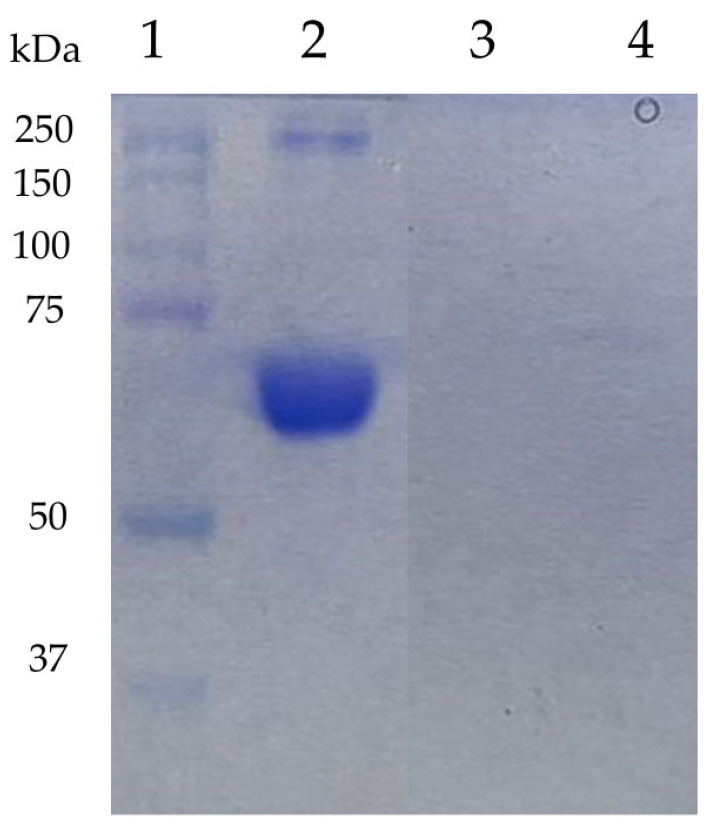
Electrophoresis conducted on the initial solutions and permeates of whey proteins, compared with a protein standard. Lane 1: Protein standard with a molecular weight (MW) of 10 to 250 kDa. Lane 2: Whey proteins in the initial solution. Lane 3: Permeate whey proteins from flat membranes with a 97% retention. Lane 4: Permeate whey proteins without ultrasound-assisted phase inversion and with 5 min of ultrasonic amplitude of 75%.

All flat membranes achieved over 97% turbidity reduction. This protein retention capacity was confirmed by gel electrophoresis (Figure A4), demonstrating effectiveness in retaining whey proteins with molecular weights between 60 and 250 kDa [70], which indicates that they all have a retention capacity of at least 60 kDa. That is, in the range for ultrafiltration separation. Huan et al. [38] reported that ultrasonically treated PVDF membranes exhibited improved filtration performance and molecular retention due to morphological changes. Under the evaluated conditions, no effect of ultrasound was observed on flat membranes’ filtration performance and retention capacity.

### 3.3. Effect of Ultrasound on the External Coagulant Bath in Hollow Fiber Membrane Fabrication

Due to technical challenges, it was not possible to replicate the experimental design to apply the same ultrasonic amplitude to both flat and hollow fiber membranes. Additionally, achieving homogeneous and well-defined membranes at amplitudes >20% proved difficult. As a result, the study of the impact of applied amplitude on the ultrasound-assisted phase inversion process for hollow fiber membranes was limited.

#### 3.3.1. Hollow Fiber Membranes Internal Structure Micrographs

**Figure 8:**
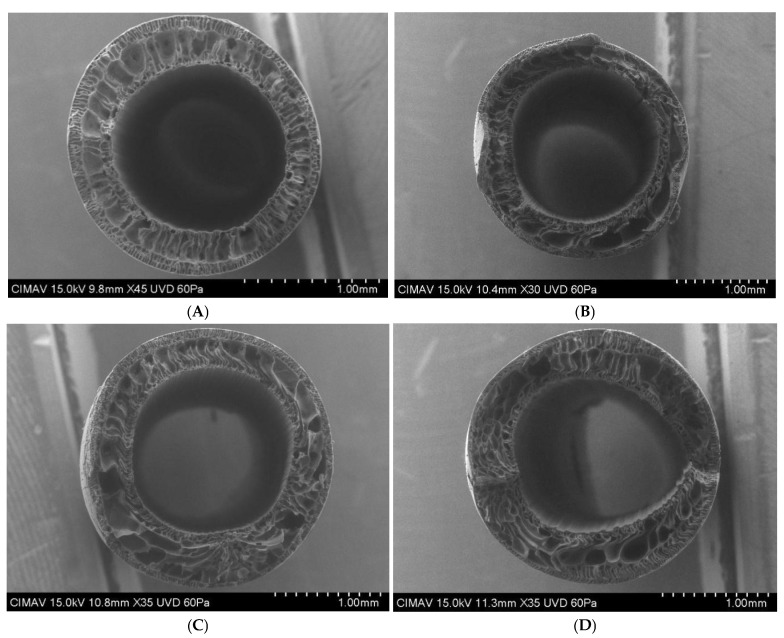
Micrographs of hollow fiber membranes with (**A**) 0% (control membrane); (**B**) 5%; (**C**) 10%; (**D**) 20% of amplitude ultrasound effect.

Scanning electron microscopy (SEM) micrographs (Figure 8) revealed the internal structure of hollow fiber membranes produced at different amplitudes. The internal structure comprised a thin active layer for separation, followed by macropores, and a second layer with external pores limited by macropores. This double-layer, finger-like structure results from rapid phase inversion using water as a non-solvent [71]. Ultrasound at 10% and 20% amplitudes expanded macropores, indicating morphological changes in hollow fiber membranes.

Cross-sections revealed higher ultrasound intensities correlated with larger macropores and reduced structural uniformity. The formation of larger macropores was directly proportional to the ultrasonic intensity applied. These alterations in the membranes exposed to ultrasound appear to be caused by the impact of sound waves on the internal structure, primarily due to the position at which they were received. The membrane subjected to 20% amplitude did not achieve concentricity, possibly due to the transition of the membrane from a liquid to a solid state during phase inversion, making it susceptible to external forces [72]. Porous materials absorb acoustic energy, causing wave collisions that enlarge macropores and displace coagulants [73]. This study is the first to apply ultrasound during hollow fiber membrane phase inversion, recommending ultrasonic application with a maximum intensity of 3.55 W/cm^2^ (20% amplitude).

#### 3.3.2. Whey Protein Filtration Capabilities of Hollow Fiber Membranes

**Figure 9:**
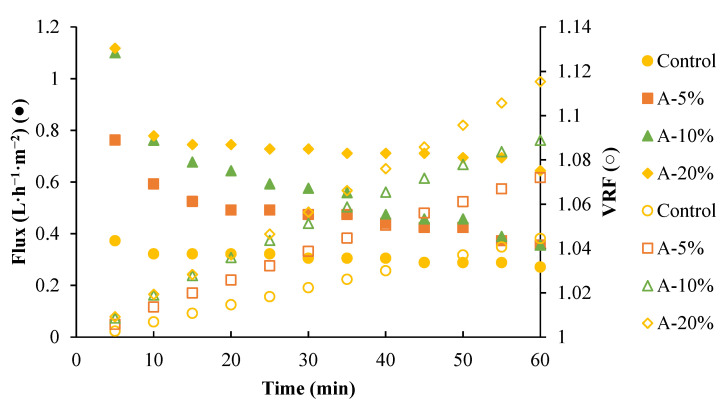
Flux and volumetric reduction factor (VRF) comparisons for whey protein filtration with hollow fiber membranes. A: ultrasound amplitude (%).

The membranes’ permeability varied according to the application of ultrasound; Figure 9 depicts filtration performance at 1 bar. Untreated membranes had low permeability (0.3 ± 0.01 L·h^−1^·m^−2^, VRF = 1.04). Ultrasonically treated membranes increased permeability as the amplitude rose, reaching 0.7 ± 0.02 L·h^−1^·m^−2^ and VRF = 1.11.

Increased filtration capacity was attributed to ultrasound-induced porosity and macrospace changes. Turbidity reduction post-filtration was measured (Table 6). Initial turbidity So (2% solution proteins) was 920 NTU. Ultrasound reduced protein retention from 90% (0% or control, 5%, 10% amplitude), while there was only 70% protein retention when applying an amplitude of 20%, where larger pores increased permeability but decreased retention. Control membranes exhibited higher flux stability and whey protein retention (90.57 ± 0.914), indicating smaller pore sizes relative to proteins and that the tangential filtration of hollow fiber membranes, unlike the cross-flow of flat membranes, could prevent the formation of any fouling [73,74,75]. In the A-20% membrane, a permeability loss of 42.42 ± 1.24% was observed, with no fouling formation, primarily due to the increase in pore size caused by ultrasound. This was confirmed by the low protein retention (70.08 ± 2.42%), allowing proteins to pass through easily without blocking the pores. Initially, the membrane showed higher flux, but as filtration progressed, some proteins and ions in milk protein solutions, such as sodium, zinc, and calcium, adhered, reducing permeability as reported by Wang et al. [68,76]. Given the excellent dispersion in the protein retention capacity of these hollow fiber membranes, HPLC analysis was performed to determine the MWCO of each membrane obtained.

#### 3.3.3. Hollow Fiber MWCO

**Figure 10:**
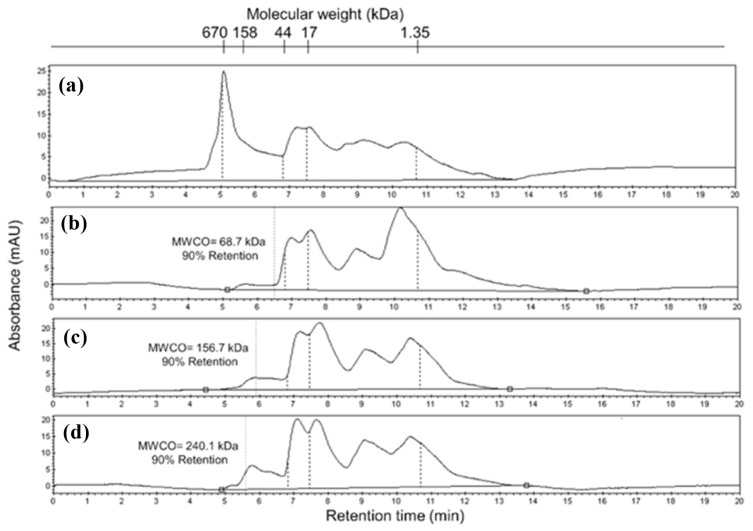
Chromatographic profiles of UV protein signal at 254 nm. (**a**) Protein solution fed to determine the molecular weight cut-off (MWCO); (**b**) 0%, (**c**) 5%, and (**d**) 20% of ultrasonic amplitude applied during phase inversion.

The membrane’s molecular weight cut-off (MWCO) determination was performed by comparing the area under the curve determined in the chromatogram (Figure 10) of the fed solution (So) and the 10% in the permeate obtained from each of the membranes in which ultrasonic amplitudes of 0, 5, 10, and 20% were applied during phase inversion. The retention time (RT) of 10% of the initial area of the protein fraction in the permeate was identified (corresponding to 90% retention). The MWCO of the membrane was estimated using the equation obtained from a calibration curve (LogMW = −0.6174 RT + 8.8441; r^2^ = 0.97) with the molecular weight of the markers (RT670 kDa = 5.05 min; RT158 kDa = 5.62 min; RT 44 kDa = 6.71 min; RT 17 kDa = 7.47 min; RT 1.35 = 110.69 min).

The chromatographic profiles (Figure 10) clearly show that the membrane without US treatment has higher retention, and as the ultrasonic amplitude in the external coagulant bath increases, lower retention of low MW proteins is observed, i.e., the MWCO of the membranes increases. This paper reports the effect of a range of ultrasonic amplitudes during ultrasound-assisted phase inversion using an ultrasonic probe, thereby expanding the technological bases to carry out studies aimed at the preparation of membranes with ultrasonic pulses during phase inversion. Further studies are being performed in our research group on this effect to expand this knowledge.

## 4. Conclusions

These results confirm the hypothesis about the efficient use of pulse using ultrasonic probes connected to high-power generators to accelerate the dissolution of polymer blends and improve the performance of phase inversion membranes. It was established in this system how to significantly reduce polymer dissolution time without compromising viscosity, thermal stability, or rheological behavior, which was beneficial for polymer dissolution using diacetin as a green co-solvent. Ultrasound-assisted phase inversion improved the ultrafiltration permeability of flat membranes and scattered the retention capacity of hollow fiber membranes, observing a direct relationship between the applied ultrasonic amplitude and the increase in MWCO. This technique effectively produced flat and hollow fiber membranes with MWCO for ultrafiltration applications, which allows the separation, purification, and/or concentration of compounds of interest in fluid foods and agro-industrial effluents such as proteins, peptides, and dyes, among others, which, if not removed, become environmental pollutants. However, further studies are still needed on this system, for example, on the effects of changing the MWCO of the additives on the structure and properties of the membranes, as well as the possible incorporation of composites or new biopolymers to produce functionalized membranes prepared by ultrasound-assisted phase inversion using ultrasonic probes with high-power generators. These perspectives are in order to achieve membranes with better properties and greater targeted applications.

## Figures and Tables

**Table 1:** Effects of ultrasound on polymeric mixtures’ dissolution and flat membranes’ production by ultrasound-assisted phase inversion in ultrasonic baths.

Composition (Polymer/Additive/Solvent)	Process US	Conditions (kHz/Hzmax/W/Wmax)	Application Time	Observations	Reference
PVDF/LiCl/DMF	Phase inversion	40 kHz/40 kHz/180–300 W/300 W	60 s	Improves interdiffusion between solvent and nonsolvent, affecting membrane morphology and performance.	[36]
PVDF/NMP	Phase inversion	45, 80, 100 kHz/100 kHz/120–300 W/300 W	20 min	Promotes the formation of cellular pores and improves membrane porosity and permeability.	[37]
PVDF/MWCNT/DMF	Phase inversion	Not reported/100 kHz/60 W, 80 W, and 100 W/300 W	20 s, 40 s, and 60 s	Modified membrane structure by promoting the formation of finger-like pores instead of macropores. Improved permeability.	[38]
CA/ZnO/DMF	Phase inversion	Not reported	8 h	Increases pore size, improves hydrophilicity, and enhances mechanical stability.	[10]
CTA/Graphene oxide/DMF	Polymer disolution	Not reported	90 min	Facilitates graphene dispersion in the polymer matrix, improving structural orientation.	[39]
CA/PEG-400, PS, or PL/DMAc	Polymer disolution	Not reported	Not reported	Aids in the dissolution of additives in the polymer solution promote homogeneity.	[40]
PVDF/PVP/DMF	Degassing polymer solution	Not reported	10 min	Allowed the removal of bubbles from the polymer mixture.	[41]

(CA) Cellulose acetate; (CTA) Cellulose triacetate; (PVDF) polyvinylidene fluoride; (DMF) dimethylformamide; (MWCNT) multiwalled carbon nanotubes; (PEG) polyethylene glycol; (PS) polysulfone; (PL) Pluronic F127; (DMAc) N,N-dimethylacetamide; (PVP) polyvinylpyrrolidone.

**Table 2:** Effect of ultrasonic intensity on polymer blend dissolution.

Polymer Blends	Ultrasonic Intensity (W/cm^2^)	Effective Dissolution Time (min)	Apparent Viscosity (Pa·s)
PEG-CA	0 ± 0.00 ^a^	2880 ± 30.01 ^a^	3.24 ± 0.06 ^ab^
PEG-CA-A50%	59.61 ± 3.89 ^b^	35.0 ± 2.50 ^b^	3.17 ± 0.04 ^a^
PEG-CA-A100%	123.23 ± 4.19 ^c^	17.5 ± 1.01 ^b^	3.43 ± 0.02 ^b^
PEG-A50% + CA	55.81 ± 4.6 ^b^	32.5 ± 2.50 ^b^	3.23 ± 0.03 ^ab^
PEG-A100% + CA	118.6 ± 4.00 ^c^	15.0 ± 1.01 ^b^	3.25 ± 0.07 ^ab^

(PEG) polyethylene glycol; (CA) cellulose acetate; (A) US amplitude. The results are expressed as the mean ± standard deviation (n = 3). Different letters by columns represent statistically significant differences (*p* < 0.05).

**Table 3:** Rheological parameters of the Cross model for stirred and sonicated polymer blends.

Blends	R^2^	$\eta_{0}$ (Pa·s)	$\eta_{\infty }$ (Pa·s)	*λ* (s)	*n*
PEG-CA	0.974 ± 0.001	4.25 ± 0.25	0.73 ± 0.26	0.002 ± 0.0002	0.381 ± 0.169
PEG-CA-A50%	0.978 ± 0.014	3.55 ± 0.13	0.18 ± 0.04	0.002 ± 0.0001	0.257 ± 0.200
PEG-CA-A100%	0.987 ± 0.010	3.88 ± 0.19	0.21 ± 0.12	0.002 ± 0.0001	0.371 ± 0.141
PEG-A50%-CA	0.996 ± 0.001	4.32 ± 0.06	0.17 ± 0.01	0.002 ± 0.0001	0.444 ± 0.323
PEG-A100%-CA	0.988 ± 0.002	3.69 ± 0.08	0.21 ± 0.33	0.002 ± 0.0002	0.371 ± 0.202

The results are expressed as the mean ± standard deviation (n = 3).

**Table 4:** Weight loss temperatures and residue for polymer blends under maximum ultrasonic intensity, including endothermic transitions.

Polymer Blends	T 80% (°C)	T 90% (°C)	T 95% (°C)	Glass Transition Temperature T_g_ (°C)	Melting Temperature T_m_ (°C)
PEG-CA	176.50 ± 2.12 ^a^	321.5 ± 2.12 ^a^	399.0 ± 2.41 ^a^	165.98 ± 0.06 ^a^	217.56 ± 1.35 ^a^
PEG-CA-A100%	172 ± 1.41 ^a^	302.5 ± 3.53 ^b^	397.0 ± 1.70 ^a^	166.07 ± 0.46 ^a^	225.52 ± 1.49 ^b^
PEG-A100%-CA	164 ± 1.41 ^b^	292.5 ± 1.53 ^c^	395.5 ± 1.94 ^a^	165.63 ± 0.27 ^a^	220.78 ± 1.47 ^a^

The results are expressed as the mean ± standard deviation (n = 3). Different letters indicate statistically significant differences.

**Table 5:** Reduction in turbidity and retention of flat membranes.

Membrane	NTU in Permeate	Retention (%)
T0-A0	19.5 ± 0.7 ^a^	97.22 ± 0.1 ^a^
T1-A25	20.5 ± 0.7 ^a^	97.08 ± 0.1 ^a^
T3-A25	19.5 ± 0.7 ^a^	98.22 ± 0.1 ^a^
T5-A25	20.5 ± 0.7 ^a^	97.08 ± 0.1 ^a^
T1-A50	20.5 ± 0.7 ^a^	97.08 ± 0.1 ^a^
T3-A50	20.5 ± 0.7 ^a^	97.08 ± 0.1 ^a^
T5-A50	19.5 ± 0.7 ^a^	97.22 ± 0.1 ^a^
T1-A75	20.5 ± 0.7 ^a^	97.08 ± 0.1 ^a^
T3-A75	19.5 ± 0.7 ^a^	97.22 ± 0.1 ^a^
T5-A75	19.0 ± 1.4 ^a^	97.29 ± 0.2 ^a^

The results are expressed as the mean ± standard deviation (n = 3). Different letters indicate statistically significant differences. NTU: nephelometric turbidity units; T: time (ultrasound application in minutes); A: ultrasound amplitude (%).

**Table 6:** Turbidity reduction in NTU by the different membranes evaluated.

Membrane	NTU in Permeate	Reduction (%)
A-0% (Control)	86.67 ± 8.40 ^a^	90.57 ± 0.914 ^a^
A-5%	50.33 ± 6.65 ^b^	94.52 ± 0.72 ^b^
A-10%	103.67 ± 3.05 ^a^	88.72 ± 0.33 ^a^
A-20%	275.00 ± 22.27 ^c^	70.08 ± 2.42 ^c^

The results are expressed as the mean ± standard deviation (n = 3). Different letters indicate statistically significant differences. A: Ultrasound amplitude. Initial turbidity of 920 NTU (nephelometric turbidity units) of 2% whey protein solution.

## Data Availability

The original contribution presented in the study is included in the article; further inquiries can be directed to the corresponding authors.

## Abbreviations

CA	cellulose acetate
PES	polyethersulfone
PSF	polysulfone
PVDF	polyvinylidene
PP	polypropylene
NIPS	non-solvent-induced phase separation
PVP	polyvinyl pyrrolidone
PEG	polyethylene glycol
MWCO	molecular weight cut-off
NMP	N-methyl-2-pyrrolidone
DMAc	dimethylacetamide
DMF	N,N-dimethylformamide
TGA	thermogravimetric analysis
DSC	differential scanning calorimetry
SEM	scanning electron microscope
J	permeate flux (L·h^−1^·m^−2^)
R	protein retention capacity
NTU	nephelometric turbidity unit
VRF	volume reduction factor
SDS	sodium dodecyl sulfate
G′	storage modulus
G″	loss modulus
T_g_	glass transition temperature
T_m_	melting temperature
MW	molecular weight
